# Use of air stacking to improve pulmonary function in Indonesian Duchenne muscular dystrophy patients: bridging the standard of care gap in low middle income country setting

**DOI:** 10.1186/s12919-019-0179-4

**Published:** 2019-12-16

**Authors:** Kristy Iskandar, Sunartini, Andika Priamas Nugrahanto, Nissya Ilma, Alvin Santoso Kalim, Guritno Adistyawan, Siswanto, Roni Naning

**Affiliations:** 1 Department of Child Health/Genetics Working Group, Faculty of Medicine, Public Health and Nursing/UGM Academic Hospital, Universitas Gadjah Mada, Jl. Kabupaten (Lingkar Utara), Kronggahan, Trihanggo, Gamping, Sleman, Yogyakarta, 55291 Indonesia; 2 Genetics Working Group, Faculty of Medicine, Public Health and Nursing, Universitas Gadjah Mada, Yogyakarta, Indonesia; 3 Department of Child Health, Faculty of Medicine, Public Health and Nursing, Dr. Sardjito Hospital, Universitas Gadjah Mada, Yogyakarta, Indonesia; 4 Department of Rehabilitation and Physical Medicine, UGM Academic Hospital, Yogyakarta, Indonesia; 5 Department of Pulmonology and Respiratory Medicine, UGM Academic Hospital, Yogyakarta, Indonesia

**Keywords:** Respiratory devices, Duchenne muscular dystrophy, Respiratory function

## Abstract

**Background:**

Duchenne Muscular Dystrophy (DMD) is a fatal X-linked recessive neuromuscular disease, characterized by progressive loss of muscle strength. Respiratory failure is the main cause of morbidity and mortality in DMD patients. Respiratory devices have been reported to increase the effectiveness of cough and pulmonary function, thus prolong the survival rate. However, there is scarcity of studies about DMD patients’ respiratory profiles and usage of respiratory devices in Indonesia.

**Methods:**

We recruited 8 Indonesian DMD patients in Dr. Sardjito Hospital and UGM Academic Hospital, Yogyakarta. Baseline pulmonary function was measured using spirometry. Peak Cough Flow was measured at baseline, with chest compression, after air stacking with manual ventilation bag, and with the combined techniques. Data recorded was presented as mean ± SD and analysed using ANOVA.

**Results:**

Here we show the respiratory profiles from 8 non-ambulatory DMD patients (mean age: 13.25 ± 3.96 years old) confirmed by genetic testing. None of them had access to respiratory devices. Spirometry measurements showed 7 of 8 patients had severe restrictive pulmonary function with mean FEV_1_/FVC 22.40 ± 10.30% of predictive values (normal ratio > 70%). In addition, all patients showed poor cough performances measured by peak cough flowmeter (160 ± 44.58 L/min (normal value > 270 L/min)) that were improved by air stacking using a manual ventilation bag (167.4 ± 46.72 L/min). Three patients who had nocturnal hypoventilation did not have daytime hypercapnia. Manual ventilation bag or mechanical in−/ex-sufflation was indicated in 75% of patients while nocturnal assisted ventilation was indicated in 50% of patients. Neither daytime assisted ventilation nor tracheostomy was indicated in these patients.

**Conclusion:**

Use of manual exsufflation in combination with the manual ventilation bag for air stacking to improve cough performance is recommended as the first step of respiratory management in DMD patients. Provision of manual ventilation bag serve as an affordable and effective device for respiratory support in the early stage of respiratory involvement in those non-ambulatory patients with DMD.

## Background

Duchenne muscular dystrophy (DMD) is an X-linked recessive progressive neuromuscular disease caused by DMD gene mutation which encodes dystrophin, a large cytoskeletal, structural protein for muscle membrane stability. If left untreated, muscular dystrophies will compromise respiratory function resulting in lower upper airway tone, abnormal chest wall compliance and weak inspiratory muscle. This condition can cause ineffective ventilation leading to chronic respiratory insufficiency [1].

Altered cough efficiency in DMD patients, due to neuromuscular weakness that affects the inspiratory and expiratory muscles causing glottis dysfunction, is aggravated by scoliosis [2]. Respiratory failure frequently occurs due to impaired secretion clearance, mucus plugging, and inadequate cough during upper respiratory tract infection that eventually requires continuous ventilation support until premature death [3].

In the early non-ambulatory stage, DMD patients are recommended to perform lung function tests twice yearly and a sleep study using capnography for detecting obstructive sleep apnoea or sleep disorder breathing. Lung volume recruitment is necessary when FVC ≤60% of the predicted level to maintain respiratory function [4]. To prevent respiratory complications due to ineffective cough, the next respiratory intervention is to initiate cough assistance manually or mechanically in DMD patients with FVC <50% predicted, or PCF < 270 L/min or MEP < 60 cmH_2_O*.* Patients with breathing irregularity in their sleep study need nocturnal assisted ventilation. Meanwhile, patients with daytime SpO_2_ < 95%, pCO_2_ > 45 mmHg, or symptoms of awake dyspnoea require the addition of assisted daytime ventilation [4, 5].

Peak cough flow (PCF), one of the cough effectiveness parameters commonly used in clinical settings showed that PCF < 160 L/minutes is associated with ineffective cough [6]. Numerous techniques ranging from manual to mechanically assisted manuveurs of cough augmentation with varying costs have been reported to increase the effectiveness of cough and respiratory function. Air stacking with a manual ventilation bag (i.e resuscitatior bag) can increase respiratory tract clearance and cough effectiveness. This procedure is simple and available in any setting. Its effectiveness is comparable to an in-exsufflation machine which has higher cost and may not be accessible [7].

Lung function tests are needed to be routinely performed as the basis of appropriate respiratory device selection in DMD patients with compromised respiratory function. Training and practice of respiratory tract clearance at an earlier age, will decrease respiratory failure, pneumonia, and death at young age [8]. Despite the relevance of the problem, however, numerous techniques and cough augmentation methods have not been used with Indonesian DMD patients due to a lack of availability and understanding of the treatment interventions. The purpose of the present study was to measure respiratory profile in non-ambulatory DMD patients, asses the need of appropriate respiratory devices and investigate the effectiveness of cough augmentation techniques.

## Methods

This pilot study was performed at the Universitas Gadjah Mada Academic Hospital, Yogyakarta, Indonesia. Eleven patients with confirmed DMD diagnosis were enrolled. Genetic testing using multiplex ligation-dependent probe amplification (MLPA), or immunohistochemistry from muscle biopsy were used to confirm the mutation of DMD gene or absence of dystrophin. Initial assessments included anamnesis on age of initial symptoms, age at diagnosis, age at first walk, history of steroid treatment and history of immunization. Physical examination including measurement of body mass index and cobbs angle based on chest x-ray were obtained.

The inclusion criteria were non-ambulant DMD patients, age > 9 y, PCF *<* 270 L/minutes, hemodynamically stable, and absence of respiratory tract infection in the past month. We chose children who will be competent enough to understand the procedure. The exclusion criteria for air stacking method were previous lung disease, respiratory infection on the day of the assessment, history of pneumothorax, and history of cough assisted device usage. The criteria for cancellation of treatment were rejection by the patient or family, fatigue, hyperventilation, worsening of oxygenation, and barotrauma. Out of 11 patients, 3 patients did not fulfil the inclusion and exclusion criteria and therefore 8 patients were enrolled.

After the clinical evaluation, we measured baseline pulmonary function including forced vital capacity (FVC) and forced expiratory volume (FEV_1_) using Cosmed Pony FX® desktop spirometer in a seated position. Data of daytime respiratory status and sleep monitoring respiratory status of oxygen saturation, TcCO2 and heart rate were obtained. Nocturnal hypoventilation was defined based on criteria established by the American Academy of Sleep Medicine Guidelines.

Peak cough flow (PCF) was measured in all subjects at baseline (unassisted cough), with chest compression (Heimlich maneuver), after air stacking with resuscitator bag, and with the combined technique. The chest compression were performed by asking the patients to inhale deeply and hold their breath. After that step the examiner applied chest compression by giving external pressure over the rib cage when the patient exhale forcefully. For the air stacking, it involves the below steps: (1) insufflate air into resuscitator bag, an ask the patient not to exhale but to inhale deeply and hold their breath, (2) repeat the above air stacking for two more times, (3) ask the patient to cough. The face mask of the resuscitator bag was put over the patient’s face. A complete air stacking manuever consists of three insuflation without exhalation.

All measurement were made with the patient seated and taken by the same respiratory therapist. The PCF measurements were made using a disposable mouthpiece attached to a peak flow meter (Philips Respironics® Peak Flow Meter) and the resuscitator bags (Ambu® SPUR® II disposable resuscitator) which were donated by Prof Masafumi Matsuo, Kobe Gakuin University, Japan.

This study was approved by the Medical and Health Research Ethics Committee Faculty of Medicine, Public Health and Nursing Universitas Gadjah Mada (KE/FK/0838/EC/2018). Purpose and methods of the research, the expected effects, the possibility of adverse events and patients’ confidentiality were explained before completing informed consent forms.

Data were processed and analysed using ANOVA followed by Tukey’s post hoc tests. Data were presented as mean values with standard deviation (SD) (standard error of means on figures) and the 95% confidence interval. Significance was set at *p* < 0.05.

## Results

### Clinical data

We obtained data from 8 subjects diagnosed with DMD; DMD gene deletions were found in 6 subjects, while 2 patients had absence of dystrophin showed by immunohistochemistry from muscle biopsy. The mean of CK level was 5056.88 ± 4243.63 IU/L (normal reference: 0–200 IU/L). Clinical characteristics of the subjects are shown in Table 1.

**Table 1:** Patient characteristic

Characteristic	n
Total number of patients	8
Age, y	13.25 ± 3.96
Weight, kg	27.48 ± 5.50
Height, cm	148.99 ± 7.28
BMI, kg/m^2^	12.46 ± 2.85
Genetic	
Deletion	6 (75%)
Duplication	0 (0%)
Point mutation	0 (0%)
Undefined mutation	2 (25%)
Age of Initial Symptom, y^a^	3.92 ± 2.27
Age when Diagnosed, y	5.12 ± 3.02
Age at First Walk, y	1.39 ± 0.35
Scoliosis	
Mild (<20^o^)	2 (25%)
Moderate (20^o^ < x < 40^o^)	3 (37.5%)
Severe (>40^o^)	3 (37.5%)
Spinal Fusion	0 (0%)
CK Level	5056.88 ± 4243.63
Family History^b^	
Yes	6 (75%)
No	2 (25%)
Steroid Treatment	
Yes	5 (62.5%)
No	1 (12.5%)
Paused	2 (25%)
Immunization^c^	
Yes	0 (0%)
No	8 (100%)

^a^Initial symptom: first onset of motoric symptoms complained by the subject parents. Symptoms were included frequent falling, motoric regression, tiptoe walking, waddling gait or gower sign

^b^Three generation family member with the same symptom or diagnosed as genetic neuromuscular disease

^c^Influenza and PCV Immunization

All subjects use wheelchairs in their daily activities. Furthermore, 5 of 8 subjects had been using steroid treatment and 1 subject had never used steroids. X-ray examinations showed all subjects had mild to severe scoliosis (reference range: mild scoliosis (<20^o^), moderate scoliosis (20^o^ < x < 40^o^), and severe scoliosis (>40^o^)).

### Pulmonary function

Pulmonary function was measured from the subjects using spirometry. One subject was unable to perform FEV_1_ due to intellectual incapacity to understand and perform the test. The results of pulmonary function tests are reported in Table 2. During the study, the mean FVC of the subjects was 1005 ± 446.51 L (range from 420 to 1660) with the % predicted value for FVC being 40.25 ± 20.91%, whereas the mean FEV_1_/FVC was 82.4 ± 24.0%.

**Table 2:** Pulmonary function

Respiratory characteristic	Mean ***±*** SD
FVC (L)	1005 ± 446.51
FVC (% pred)	40.25 ± 20.91
FEV_1_ (L)	765.71 ± 432.31
FEV_1_ (% pred)	32.91 ± 22.54
FEV_1_/FVC (%)	82.42 ± 24.00
Nocturnal Respiratory Status	
SpO_2_	
Most common SpO_2_ (%)	96.5 ± 1.19
Lowest SpO_2_ (%)	91.13 ± 5.33
Duration of SpO_2_ < 90% (%)	1.09 ± 1.85
Duration of SpO_2_ < 90% (min)	5 ± 8.87
pCO_2_	
Most common pCO_2_ (mmHg)	31.13 ± 5.08
Highest pCO_2_ (mmHg)	36.5 ± 3.42
pCO_2_ > 45 mmHg (%)	0
Awake Respiratory Status	98.38 ± 1.19
SpO_2_ (%)	29.25 ± 4.86
pCO_2_(mmHg)	19.13 ± 1.88
Respiration Rate (x/minutes)	

Measurement of respiratory status with SpO2 and TcCO2 monitoring were performed at night during sleep and during daytime when awake (Table 2). During awake state the patients had better SpO2 (mean: 98 ± 1.18%) and TcCO2 (mean: 29 ± 4.86 mmHg) than during sleep at night SpO2 (mean: 96 ± 1.19%) and TcCO2 (mean: 31 ± 5.08 mmHg). Awake respiratory status showed better results than measurements taken during sleep. There was no daytime hypercapnia recorded in all of the patients (normal TcCO2 is 35–45 mmHg). Over one-third (37.5%) of the patients had nocturnal hypoventilation based on nocturnal SpO_2_ and TcCO_2_ criteria. Nocturnal hypoventilation were defined as TcCO_2_ > 55 mmHg for ≥10 min or increase in TcCO_2_ ≥ 10 mmHg (in comparison to an awake supine value) to a value exceeding 50 mmHg for ≥10 min and Mean nocturnal SpO_2_ < 90% or SpO_2_ < 90% during ≥10% of the recording time [9, 10].

**Fig. 1:**
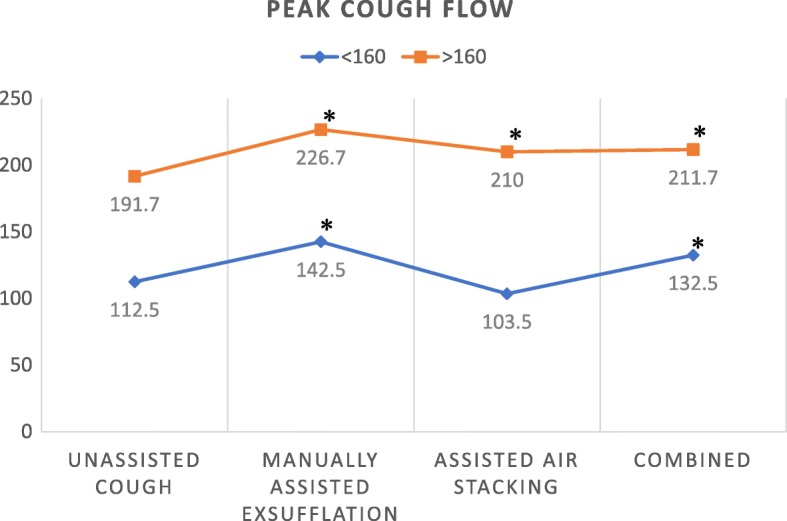
Peak Cough Flow (PCF) value at baseline, after chest compression, after air stacking using ambu bag and after combined technique (chest compression and ambu bag air stacking), PCF < 160 L/min: 2 patients, PCF > 160 L/min: 3 patients. *p-value< 0.05.

The mean PCF at baseline without any intervention, with chest compression, with air stacking and with the combined technique were 160 ± 44.58, 193 ± 46.72, 167.4 ± 46.72 and 180 ± 45.69 L/minutes, respectively (Table 3). The PCF values measured with chest compression, after air stacking and with the combined technique were significantly higher than those measured at baseline without any intervention (*p* < 0.05) (Fig. 1).

**Table 3:** PCF value

PCF value (L/min)	Mean ± SD
Unassisted Cough	160 ± 44.58
Manually Assisted Exsufflation	193 ± 46.72
Assisted Air Stacking	167.4 ± 46.72
Combined Technique	180 ± 45.69

**Fig. 2:**
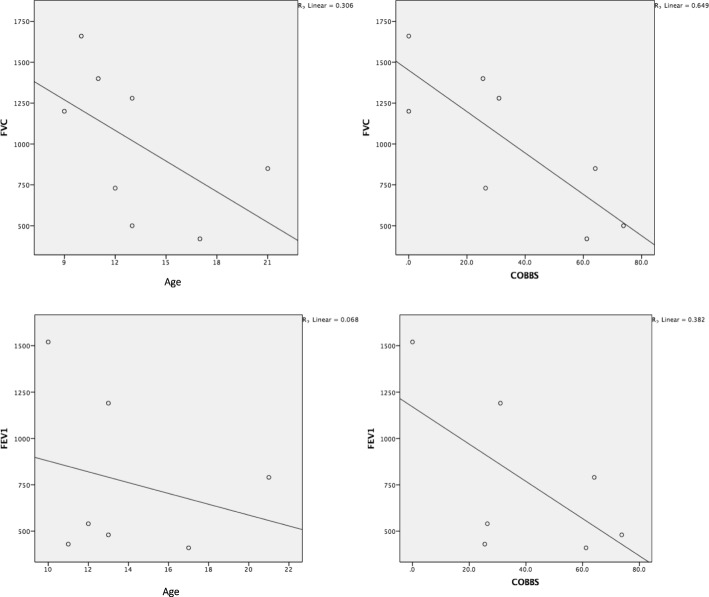
Correlation between lung function (FVC and FEV_1_) and clinical characteristic (age and degree of cobbs angle)

Baseline mean PCF for patients with pronounced (moderate to severe) scoliosis (153.33 ± 35.45 L/min) were found to be lower than the findings from patients without scoliosis (226.5 ± 37.47 L/min). In regards to the enhancement of the PCF in the chest compression technique, a noticeable difference was found between patients with scoliosis and those without. (*p* = 0.016) (Fig. 2).

## Discussion

In this study, in those subjects who have mean PCF at baseline > 160 L/min, improvement was noted after the application of air-stacking, chest compression and the combined technique. As the indication of assisted coughing is mean PCF less than 270 L/min, all our patients need to have intervention to assist their coughing. A DMD patients’ mean PCF is likely to fall below 160 L/min with viral infection [6]. In this study, our patients at a later stage of respiratory involvement with mean PCF less than 160 L/min could not have their mean PCF improved by manually assisted coughing maneuvers.

Previous reports showed peak cough flow with air stacking had varying magnitudes of improvement between 19 and 87% [11], perhaps because of disparities in samples, baseline lung functions, the distinct air stacking techniques being used, and the therapist’s expertise. Discordance of technique performance between the patient and the respiratory therapist, air leakage from the mask, as well as insufficient force or improper application of chest compression are some of the problems that are encountered with the manually assisted cough technique. In consequence, thorough and simple training for patients and caregivers is essential so that they can do the proper maneuvers without the aid of health care professionals [12]. In this study, it is believed that the data has not been negatively affected by improper technique application because air stacking has always been conducted by the same respiratory therapist.

The American Thoracic Society (ATS) (2004) recommends the use of cough assistance device for patients with PCF less than 270 L/min [13]; thus 75% of subjects included in our study were already in need of manual ventilation bag or mechanical in−/ex-sufflation. These patients had ineffective cough which is important for them to use manual exsufflation and can combine the use of ventilation bag for air stacking to improve cough effect to remove excess secretions from the airways thus decreasing the risk to develop respiratory infection, atelectasis, and respiratory failure. Nocturnal assisted ventilation was indicated in 50% of our patients following the recommendation of ATS when there are abnormal results from the sleep study and the FVC is less than 50% predicted [13]. Failure in early identification and treatment of nocturnal hypoventilation in DMD patients will result in their quality of life and premature mortality. None of our patients in this study needed daytime assisted ventilation or tracheostomy during the study period.

One of the limitations of this study is the relatively small number of recruited subjects. Young children were not been included in this research due to their inability in performing voluntary manuvers such as spirometry reliably which would make the parameters unavailable for analysis.

Despite the essential need for respiratory support for the recruited patients, none of the patients enrolled in this study had access to any respiratory devices. Among DMD patients in the early stage of the respiratory involvement, the use of manual exsufflation and affordable air stacking method (cost 20–50 Euros) can be very helpful. At the later stage of the disease, the patients will eventually need ventilation support which costs at least 3000 Euro [7]. Seasonal influenza and pneumococcal vaccination are also important as part of the standard of care in respiratory management of DMD patients, however in Indonesia this vaccination is not yet routinely available to DMD patients and many patients and caregiver have limited knowledges on this.

In low-middle income country (LMIC) such as Indonesia, economic constraints and medical health system barriers are among the major challenges encountered by patients and medical personnel. Economic constraints posed challenges to provide standard of care for DMD patients. The challenges faced by the DMD families include unable to affort medical cost, knowledge gap and difficulty to access health care services due to geographical condition. The current barriers faced by the health care providers are limited accessibility to funding to support genetic testing for DMD diagnosis, limited skilled health care workers experienced in DMD, and the high cost of ventilation device not covered by Indonesian Universal Health Insurance. Currently, there is no national guideline in Indonesia for the diagnosis and management in children with Duchenne muscular dystrophy. Most patients do not routinely visit the outpatient clinic after first diagnosis due to the aforementioned reasons, thus adherence to steroids, with routine cardiac and lung function tests are not performed in Indonesian DMD patients.

## Conclusion

In conclusion, respiratory function needs to be evaluated periodically. Accessibility to manual exsufflation and ventilation bag for air stacking for the combined technique allows the improvement of the mean PCF and helps to maintain respiratory function and enhance recovery after respiratory infection. It is an affordable and effective approach for patients in the early stage of respiratory involvement when they reach the non-ambulatory condition. In LMICs and developing countries such as Indonesia concerns about respiratory management of non-ambulatory DMD patients should be raised with the appropriate authorities.

## Data Availability

All data generated or analysed during this study are included in the submission. The raw data are available from the corresponding author on reasonable request.

## Abbreviations

DMD: Duchenne muscular dystrophy
FEV_1_: Forced expiratory volume
FVC: Forced vital capacity
LMIC: Low-middle income countries
PCF: Peak cough flow
